# Knowledge, attitudes, and practices regarding the prevention of intracerebral hemorrhage among hypertensive patients

**DOI:** 10.3389/fcvm.2025.1361273

**Published:** 2025-06-11

**Authors:** Qiang Chen, Feng Liu, LingLing Zhang, Yang Jin, Haibin Huang

**Affiliations:** ^1^ Department of Neurosurgery, Nantong Third People’s Hospital, Affiliated Nantong Hospital 3 of Nantong University, Nantong, Jiangsu, China; ^2^ Department of Neurosurgery, Dongtai People’s Hospital, Dongtai, Jiangsu, China; ^3^ Department of Emergency, Nantong Third People’s Hospital, Affiliated Nantong Hospital 3 of Nantong University, Nantong, Jiangsu, China; ^4^ Medical Oncology, Nantong Tumor Hospital, Nantong, Jiangsu, China; ^5^ Department of Emergency, The Third Affiliated Hospital of Guangzhou University of Chinese Medicine, Guangzhou, China

**Keywords:** knowledge, attitude, practice, medication adherence, hypertension, intracerebral hemorrhage, cross-sectional study

## Abstract

**Objective:**

The present study aimed to assess knowledge, attitudes, and practices (KAP) regarding the prevention of intracerebral hemorrhage among hypertensive patients and medication adherence to hypertension.

**Methods:**

We conducted a cross-sectional study at the Third People's Hospital of Nantong City between November 4, 2023 and December 4, 2023. Demographic information, KAP and medication adherence scores were collected using an online questionnaire.

**Results:**

Totally 600 valid questionnaires were analyzed. Among these, 443 participants (73.83%) were female, with a mean age of 62.95 ± 15.07 years. The mean scores for knowledge, attitude, practice, and medication adherence were 13.93 ± 2.88, 32.10 ± 3.22, 25.53 ± 3.44, and 3.12 ± 1.85, respectively. Only 3.50% had high medication compliance to hypertension. Multivariate analyses revealed that uncertain about family history of hypertensive intracerebral hemorrhage [OR = 0.378, 95%CI: (0.218–0.656); *P* = 0.001], no smoking [OR = 4.603, 95%CI: (1.954–10.845); *P* < 0.001], and no alcohol consumption [OR = 3.522, 95%CI: (1.764–7.033); *P* < 0.001] were independently associated with proactive practice. Structural equation modeling (SEM) results revealed direct effects between knowledge and attitude (*β* = 0.999, *P* < 0.001), knowledge and practice (*β* = 1.103, *P* < 0.001), as well as attitude and practice (*β* = 0.452, *P* < 0.001).

**Conclusion:**

Hypertensive patients demonstrated sufficient knowledge, positive attitudes, and inactive practices towards preventing intracerebral hemorrhage, coupled with poor medication adherence to hypertension. Developing targeted interventions to address these gaps and promoting a holistic approach is crucial to improving overall patient outcomes in clinical practice.

## Introduction

Intracerebral hemorrhage (ICH) constitutes a significant portion, ranging from 12% to 20%, of the global stroke incidence (1). This specific type of cerebral parenchymal hemorrhage arises from the spontaneous rupture of cerebral blood vessels, representing a subtype within the broader category of cerebral apoplexy. Notably, ICH accounts for 20%–30% of all cerebral apoplexy cases in China, with alarming mortality and disability rates reaching approximately 40% and 90%, respectively (2, 3). The profound impact of ICH extends beyond individual health, inflicting substantial pain and burden on families and society (4). The escalating global aging trend intensifies the adverse consequences of ICH, underscoring the urgency to address this health challenge (5). Hypertensive intracerebral hemorrhage (HICH), a prevalent subtype within the spectrum of ICH, further accentuates the gravity of the issue (6, 7). This hemorrhagic disorder not associated with trauma to the brain parenchyma exhibits its greatest incidence during the cold season, solidifying its position as a prominent health threat (8). Remarkably, HICH surpasses other stroke types in terms of mortality and disability rates, posing a formidable risk to the overall health of our nation's residents (9). The increasing prevalence of HICH underscores the critical need for targeted research and interventions to alleviate the substantial health and societal burdens associated with this condition.

Knowledge, Attitudes, and Practices (KAP) assessment serves as a structured survey tool crucial for understanding the cognitive and behavioral dimensions of hypertensive patients (10). This approach becomes particularly relevant given the necessity of self-management and prevention among individuals with hypertension (11). Despite the imperative for proactive engagement in preventive measures, there exists a discernible deficiency in patients’ knowledge regarding relevant matters. Hence, investigating the KAP of hypertensive patients becomes imperative to address the existing gaps and enhance strategies for intracerebral hemorrhage prevention.

Therefore, this study aims to investigate KAP regarding the prevention of ICH among hypertensive patients and medication adherence to hypertension. Through shedding light on these aspects, the study seeks to provide valuable insights that can help address existing gaps, ultimately enhancing preventive strategies and improving patient outcomes. The significance of this survey is underscored by its potential to guide targeted interventions and initiatives aimed at mitigating the impact of hypertensive intracerebral hemorrhage (HICH) on public health.

## Methods

### Study design and participants

The present cross-sectional study was conducted at the Third People's Hospital of Nantong City between November 4, 2023, to December 4, 2023, focusing on participants diagnosed with hypertension. The Medical Ethics Committee of the Third People's Hospital of Nantong City approved this study, and informed consent was secured from all participants. The inclusion criteria comprised hypertension with confirmed systolic blood pressure ≥140 mmHg and/or diastolic blood pressure ≥90 mmHg, age ≥18 years, any gender. Exclusion criteria encompassed individuals who, upon entering the questionnaire survey, indicated “no” on the informed consent form before the survey initiation, questionnaires demonstrating logical errors in response to trap questions, and questionnaires displaying abnormal BMI values (less than 10 or greater than 40).

### Questionnaire and quality control

The questionnaire was developed in accordance with the *Chinese Multidisciplinary Guidelines for the Diagnosis and Treatment of hypertensive intracerebral hemorrhage* and underwent a pilot test involving 24 copies. The Cronbach's *α* coefficient, assessing internal consistency, was 0.811, indicating satisfactory reliability. The finalized questionnaire comprised demographic data (gender, age, education level, etc.) and the knowledge, attitude, practice and medication adherence dimensions. The knowledge dimension encompassed 16 questions, each scored 1 and 0 for right and wrong or unclear responses, respectively, resulting in a score range of 0–16. The attitude dimension comprised 7 questions, utilizing a 5-point Likert scale from very positive to very negative (5 and 1 points, respectively), yielding a score range of 7–35. The practice dimension involved 7 questions, also utilizing a 5-point Likert scale, with values assigned from always to never (5–1 for questions 1, 3, and 7), and the remaining questions reversed in value from 1 to 5, resulting in a score range of 7–35 points. The medication adherence scale included 8 items, with 8 points maximum, where a score <6, of 6–8 and of 8 reflected poor, moderate and good adherence, respectively (12–14).

The distribution of questionnaires to study participants was conducted electronically, employing WeChat groups within various departments such as Cardiovascular Medicine, Neurology, and Geriatrics. Attending physicians played a crucial role in identifying suitable candidates for the survey. In the internal medicine outpatient department, predominantly Cardiovascular Medicine and Neurology, nurses stationed at the registration desk distributed the questionnaires to hypertensive patients as they awaited their appointments, utilizing a QR code for survey participation. All participants received pertinent training, familiarizing themselves with the questionnaire content, and were equipped to promptly address any queries raised during the survey, ensuring the quality of responses. Additionally, trap questions were strategically integrated into the questionnaire to indirectly assess the sincerity of respondents engaging in the survey.

### Statistical analysis

SPSS 22.0 was employed for statical analysis. Continuous variates were reported as mean ± standard deviation, and categorical variables as frequency and percentage. Prior to comparing continuous variables, normality tests were applied. If the data adhered to a normal distribution, a *t*-test was employed for group comparisons. In instances where normal distribution was not observed, the Wilcoxon Mann–Whitney test was utilized for group-pair comparisons. For three or more groups with continuous variables displaying normal distribution and homogeneity of variance, ANOVA was employed. In cases where normal distribution was not met, the Kruskal–Wallis analysis of variance was utilized. Univariable and multivariable logistic regression analyses were employed for identifying factors with significant associations with good knowledge, positive attitude, and proactive practice, defined as achieving a mean score in the knowledge and attitude practice dimensions of the population (15). Additionally, univariable and multivariable logistic regression analyses were carried out for determining factors with significant associations with medication adherence. Two-tailed *P* < 0.05 suggested statistical significance.

## Results

Before statistical analysis, unqualified data were excluded, including 5 questionnaires with “no” choice for informed consent, 39 questionnaires with logical errors in trap questions, 25 questionnaires with abnormal BMI of the respondents (<10/>40), and 2 questionnaires with age less than 18 years old. The remaining valid questionnaires totaled 600. Totally 443 (73.83%) of the participants were women with ages of 62.95 ± 15.07 years, and 364 (60.67%) had overweight BMI. Additionally, 411 (68.50%) had been suffering from hypertension for more than 5 years, 463 (77.17%) had a family history of intracerebral hemorrhage or hypertension, and 399 (66.50%) had been taking antihypertensive drugs for more than 5 years.

The mean knowledge, attitude, practice, and medication adherence scores were 13.93 ± 2.88, 32.10 ± 3.22, 25.53 ± 3.44, and 3.12 ± 1.85, respectively. Demographic differences in education, family history of the disease, presence of coronary heart disease, and duration of administration of antihypertensive drugs may have contributed to differences in participants’ knowledge, attitude, practice, and adherence. BMI and the presence or absence of intracerebral hemorrhage may differentiate participants’ attitudes. The presence or absence of family history of hypertension may allow for different levels of knowledge and practice. In turn, the presence or absence of diabetes was found to make a difference in attitude, practice, and compliance (Table 1).

**Table 1:** Baseline and scores for knowledge, attitudes, and practices (KAP) and compliance.

Variables	*N* (%)	Knowledge		Attitude		Practice		Compliance	
		Mean ± SD	*P*	Mean ± SD	*P*	Mean ± SD	*P*	Mean ± SD	*P*
Total	600	13.93 ± 2.88		32.10 ± 3.22		25.53 ± 3.44		3.12 ± 1.85	
Gender			<0.001		0.004		<0.001		0.349
Male	157 (26.17)	12.78 ± 3.92		31.46 ± 3.86		22.76 ± 3.56		3.24 ± 2.03	
Female	443 (73.83)	14.33 ± 2.27		32.33 ± 2.93		26.50 ± 2.81		3.08 ± 1.78	
Age	62.95 ± 15.07								
BMI			0.343		0.014		0.146		0.445
Overweight	364 (60.67)	14.02 ± 2.72		31.84 ± 3.15		25.69 ± 3.58		3.16 ± 1.84	
Not Overweight	236 (39.33)	13.79 ± 3.10		32.50 ± 3.29		25.27 ± 3.21		3.05 ± 1.86	
Education			<0.001		0.003		<0.001		<0.001
Primary School and Below	158 (26.33)	14.39 ± 2.18		31.33 ± 3.19		26.81 ± 2.81		3.84 ± 1.91	
Junior High School	151 (25.17)	14.21 ± 2.47		32.15 ± 3.13		25.29 ± 3.67		3.04 ± 1.88	
High School/Technical School	177 (29.50)	14.02 ± 2.39		32.60 ± 2.71		25.46 ± 3.13		2.52 ± 1.29	
College/Bachelor’s and Above	114 (19.00)	12.77 ± 4.30		32.32 ± 3.88		24.16 ± 3.79		3.14 ± 2.11	
Work and Living Conditions			<0.001		<0.001		<0.001		0.080
Shift work or night shift required	41 (6.83)	13.27 ± 3.56		32.37 ± 3.40		23.78 ± 3.56		3.33 ± 2.25	
Daily working hours exceed 8	82 (13.67)	12.66 ± 4.04		30.93 ± 3.45		23.16 ± 3.57		3.06 ± 2.07	
Work or living environment with noise interference	22 (3.67)	13.77 ± 2.65		30.14 ± 3.31		23.77 ± 3.50		2.16 ± 1.41	
None of the above	455 (75.83)	14.22 ± 2.48		32.38 ± 3.08		26.19 ± 3.13		3.15 ± 1.77	
Hypertension History, years			<0.001		0.014		<0.001		0.656
≤5	112 (18.67)	12.43 ± 4.27		32.62 ± 4.11		23.50 ± 3.84		3.17 ± 2.20	
>5	411 (68.50)	14.62 ± 1.49		32.32 ± 2.69		26.45 ± 2.79		3.09 ± 1.66	
Family history of hypertension			<0.001		0.660		<0.001		0.644
Yes	463 (77.17)	14.35 ± 2.20		32.06 ± 3.16		25.89 ± 3.20		3.10 ± 1.75	
No	102 (17.00)	12.44 ± 4.24		32.35 ± 3.39		24.08 ± 3.78		3.26 ± 2.22	
Uncertain	35 (5.83)	12.60 ± 3.98		31.91 ± 3.58		24.86 ± 4.36		2.97 ± 1.90	
Family history of hypertensive intracerebral hemorrhage			<0.001		0.007		<0.001		<0.001
Yes	160 (26.67)	14.34 ± 2.28		31.66 ± 3.06		26.21 ± 3.61		3.22 ± 1.91	
No	235 (39.17)	13.31 ± 3.51		31.91 ± 3.71		24.68 ± 3.67		3.41 ± 2.08	
Uncertain	205 (34.17)	14.31 ± 2.31		32.66 ± 2.62		25.96 ± 2.79		2.71 ± 1.39	
Had intracerebral hemorrhage in the past			0.176		0.030		0.268		0.161
Yes	46 (7.67)	14.48 ± 2.03		31.11 ± 3.11		26.07 ± 3.81		3.48 ± 1.94	
No	554 (92.33)	13.88 ± 2.93		32.18 ± 3.22		25.48 ± 3.41		3.09 ± 1.84	
Diabetes			0.163		0.007		0.016		0.026
Yes	110 (18.33)	14.27 ± 2.40		31.35 ± 3.30		26.24 ± 3.31		3.47 ± 1.90	
No	490 (81.67)	13.85 ± 2.97		32.27 ± 3.18		25.37 ± 3.45		3.04 ± 1.83	
Coronary heart disease			0.011		0.041		<0.001		<0.001
Yes	75 (12.50)	14.72 ± 0.81		31.39 ± 2.66		27.05 ± 2.91		3.83 ± 1.83	
No	525 (87.50)	13.81 ± 3.04		32.20 ± 3.28		25.31 ± 3.46		3.01 ± 1.83	
Smoking			<0.001		0.001		<0.001		0.571
Yes	95 (15.83)	12.45 ± 4.13		31.08 ± 3.82		21.44 ± 2.95		3.02 ± 1.96	
No	505 (84.17)	14.20 ± 2.48		32.29 ± 3.06		26.29 ± 2.95		3.14 ± 1.83	
Alcohol consumption			<0.001		<0.001		<0.001		0.471
Yes	121 (20.17)	12.98 ± 3.71		31.13 ± 3.67		22.29 ± 3.14		3.23 ± 2.01	
No	479 (79.83)	14.16 ± 2.57		32.34 ± 3.05		26.34 ± 3.01		3.09 ± 1.80	
Duration of taking antihypertensive drugs, years			<0.001		0.003		<0.001		0.042
<1	135 (22.50)	12.03 ± 4.63		31.97 ± 4.21		23.47 ± 3.88		3.08 ± 2.27	
1–3	32 (5.33)	13.00 ± 3.47		31.75 ± 3.49		22.56 ± 3.57		3.95 ± 2.10	
3–5	34 (5.67)	13.82 ± 2.94		30.24 ± 3.60		24.44 ± 3.54		2.71 ± 1.75	
>5	399 (66.50)	14.65 ± 1.33		32.33 ± 2.69		26.55 ± 2.72		3.10 ± 1.65	

The overall knowledge dimension demonstrated that only 29.50% of the participants responded correctly to the question “The age of onset for hypertensive intracerebral hemorrhage is predominantly above 50 years” (K3). Regarding the risk factors for hypertensive cerebral hemorrhage (K5), more than 90% of the patients were aware of all the factors, except hyperglycemia (82.67%) (Supplementary Table S1).

Turning to the attitude dimension, the first thing that draws attention is that, in contrast to the attitudes of the other questions, only 31.33% strongly agreed that hypertensive patients should measure their blood pressure every day (A1). In addition, 66.83% strongly agreed that exercise is important for health and disease prevention (A7). As for the remaining questions, nearly 80% of the participants were in full agreement, showing their relatively well positive attitudes (Supplementary Table S2).

The practice dimension presented the different frequency of practice of the participants, with 53.00% and 54.00% regularly eating vegetables and fruits (P2) and drinking milk (P4). 49.17% tested their blood pressure occasionally (P1). 36.17% and 48.33% occasionally ate salt or sodium-containing condiments (P3) and pickled foods (P5). In addition, 62.50% and 76.17% never drank alcohol (P6) and smoked cigarettes (P7) (Supplementary Table S3).

**Figure 1:**
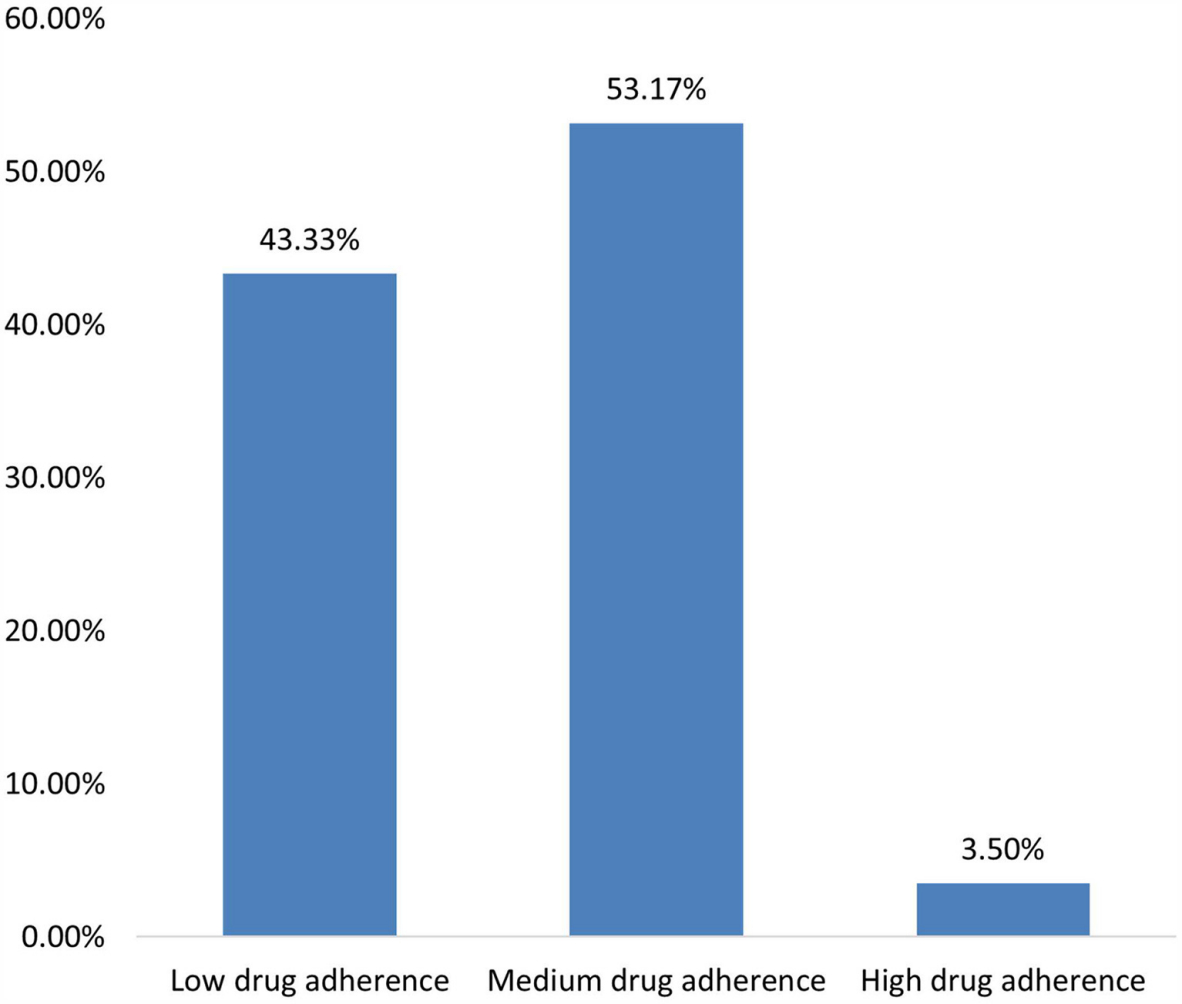
Patient compliance profile.

The patient compliance profile showed that the maximum proportion of patients (53.17%) had moderate medication compliance, followed by 43.33% whose medication compliance was low; however, only 3.50% had high medication compliance (Figure 1).

The multivariate results showed that being female [OR = 2.215, 95%CI: (1.077–4.553); *P* = 0.031], unaware of her hypertension family history [OR = 0.387, 95%CI: (0.151–0.993); *P* = 0.048], and taking antihypertensive drugs for 3–5 years [OR = 3.428, 95%CI: (1.124–10.45); *P* = 0.030] were independently associated with good knowledge (Table 2). On the other hand, the knowledge score [OR = 1.170, 95%CI: (1.085–1.262); *P* < 0.001], age [OR = 0.976, 95% I: (0.955–0.997); *P* = 0.026], graduation from high school or technical school [OR = 2.019, 95%CI: (1.122–3.631); *P* = 0.019], working more than 8 h daily [OR = 0.366, 95%CI: (0.197–0.680); *P* = 0.001], working or living with noise interference [OR = 0.288, 95%CI: (0.098–0.845); *P* = 0.023], and no coronary heart disease [OR = 1.963, 95%CI: (1.067–3.610); *P* = 0.030] had independent associations with positive attitude (Table 3). Additionally, uncertainty about family history of hypertensive intracerebral hemorrhage [OR = 0.378, 95%CI: (0.218–0.656); *P* = 0.001], no smoking [OR = 4.603, 95%CI: (1.954–10.845); *P* < 0.001], and no alcohol consumption [OR = 3.522, 95%CI: (1.764–7.033); *P* < 0.001] had independent associations with proactive practice (Table 4).

**Table 2:** Analysis of factors affecting good knowledge.

Variables	Univariate analysis		Multivariate analysis	
	OR (95%CI)	*P*	OR (95%CI)	*P*
Gender				
Male	ref.			
Female	4.754 (3.022 7.480)	<0.001	2.215 (1.077 4.553)	0.031
Age	1.061 (1.045 1.077)	<0.001	1.019 (0.992 1.046)	0.164
BMI				
Overweight	ref.			
Not overweight	0.929 (0.598 1.443)	0.743		
Education				
Primary School and Below	ref.		ref.	
Junior High School	0.538 (0.253 1.144)	0.107	1.488 (0.585 3.783)	0.404
High School/Technical School	0.419 (0.206 0.854)	0.017	1.104 (0.439 2.777)	0.833
College/Bachelor's and above	0.171 (0.084 0.347)	<0.001	1.618 (0.539 4.857)	0.391
Work and living conditions				
Shift work or night shift required	0.312 (0.150 0.648)	0.002	1.361 (0.577 3.211)	0.482
Daily working hours exceed 8	0.236 (0.138 0.403)	<0.001	0.678 (0.343 1.342)	0.265
Work or living environment with noise interference	0.439 (0.155 1.239)	0.120	0.911 (0.249 3.335)	0.889
None of the above	ref.		ref.	
Hypertension history, years				
≤ 5	ref.		ref.	
>5	9.717 (5.897 16.009)	<0.001	1.955 (0.454 8.414)	0.368
Family history of hypertension				
Yes	ref.		ref.	
No	0.183 (0.111 0.301)	<0.001	0.876 (0.445 1.726)	0.702
Uncertain	0.217 (0.101 0.463)	<0.001	0.387 (0.151 0.993)	0.048
Family history of hypertensive intracerebral hemorrhage				
Yes	ref.		ref.	
No	0.222 (0.118 0.418)	<0.001	0.667 (0.298 1.492)	0.324
Uncertain	0.919 (0.436 1.936)	0.824	0.871 (0.350 2.168)	0.766
Had intracerebral hemorrhage in the past				
Yes	ref.		ref.	
No	0.217 (0.052 0.910)	0.037	0.233 (0.046 1.184)	0.079
Diabetes				
Yes	ref.			
No	0.515 (0.265 1.000)	0.050		
Coronary heart disease				
Yes	ref.		ref.	
No	0.258 (0.092 0.725)	0.010	0.823 (0.258 2.629)	0.743
Smoking				
Yes	ref.		ref.	
No	4.360 (2.665 7.134)	<0.001	1.349 (0.538 3.380)	0.523
Alcohol consumption				
Yes	ref.		ref.	
No	3.197 (1.999 5.114)	<0.001	1.012 (0.495 2.068)	0.973
Duration of taking antihypertensive drugs, years				
< 1	ref.		ref.	
1–3	1.218 (0.551 2.692)	0.626	1.349 (0.538 3.380)	0.523
3–5	3.410 (1.325 8.779)	0.011	3.428 (1.124 10.458)	0.030
>5	11.946 (6.947 20.543)	<0.001	2.987(0.640 13.943)	0.164

**Table 3:** Analysis of factors affecting positive attitudes.

Variables	Univariate analysis		Multivariate analysis	
	OR (95%CI)	*P*	OR (95%CI)	*P*
Knowledge score	1.127 (1.058 1.200)	<0.001	1.170 (1.085 1.262)	<0.001
Gender				
Male	ref.			
Female	1.397 (0.969 2.014)	0.074		
Age	0.978 (0.967 0.989)	<0.001	0.976 (0.955 0.997)	0.026
BMI				
Overweight	ref.		ref.	
Not overweight	1.682 (1.200 2.359)	0.003	1.416 (0.964 2.079)	0.076
Education				
Primary School and Below	ref.		ref.	
Junior High School	2.167 (1.376 3.414)	0.001	1.707 (0.999 2.916)	0.050
High School/Technical School	3.092 (1.976 4.837)	<0.001	2.019 (1.122 3.631)	0.019
College/Bachelor's and Above	2.518 (1.534 4.133)	<0.001	1.751 (0.819 3.743)	0.149
Work and living conditions				
Shift work or night shift required	0.950 (0.493 1.829)	0.877	0.664 (0.302 1.462)	0.309
Daily working hours exceed 8	0.389 (0.240 0.630)	<0.001	0.366 (0.197 0.680)	0.001
Work or living environment with noise interference	0.228 (0.088 0.594)	0.002	0.288 (0.098 0.845)	0.023
None of the above	ref.		ref.	
Hypertension history, years				
≤ 5	ref.			
>5	1.208 (0.853 1.709)	0.287		
Family history of hypertension				
Yes	ref.			
No	1.258 (0.809 1.957)	0.308		
Uncertain	0.706 (0.355 1.404)	0.321		
Family history of hypertensive intracerebral hemorrhage				
Yes	ref.		ref.	
No	1.278 (0.854 1.911)	0.233	1.043 (0.628 1.733)	0.870
Uncertain	2.609 (1.695 4.016)	<0.001	1.271 (0.752 2.149)	0.371
Had intracerebral hemorrhage in the past				
Yes	ref.		ref.	
No	2.258 (1.219 4.180)	0.010	1.263 (0.610 2.615)	0.529
Diabetes				
Yes	ref.		ref.	
No	2.300 (1.509 3.506)	<0.001	1.649 (0.982 2.769)	0.059
Coronary heart disease				
Yes	ref.		ref.	
No	2.579 (1.565 4.250)	<0.001	1.963 (1.067 3.610)	0.030
Smoking				
Yes	ref.		ref.	
No	1.909 (1.226 2.970)	0.004	0.917 (0.483 1.743)	0.792
Alcohol consumption				
Yes	ref.		ref.	
No	2.109 (1.408 3.159)	<0.001	1.641 (0.922 2.920)	0.092
Duration of taking antihypertensive drugs, years				
< 1	ref.		ref.	
1–3	0.733 (0.337 1.591)	0.431	1.005 (0.416 2.430)	0.991
3–5	0.353 (0.161 0.772)	0.009	0.565 (0.224 1.424)	0.226
>5	0.926 (0.622 1.380)	0.706	1.058(0.535 2.092)	0.871

**Table 4:** Analysis of factors affecting proactive practices.

Variables	Univariate analysis		Multivariate analysis	
	OR (95%CI)	*P*	OR (95%CI)	*P*
Knowledge score	1.178 (1.092 1.270)	<0.001	1.084 (0.997 1.179)	0.058
Attitude score	1.026 (0.976 1.079)	0.309		
Gender				
Male	ref.		ref.	
Female	4.335 (2.914 6.509)	<0.001	1.428 (0.751 2.713)	0.277
Age	1.040 (1.028 1.053)	<0.001		
BMI				
Overweight	ref.		ref.	
Not Overweight	0.704 (0.507 0.978)	0.037	1.022 (0.998 1.047)	0.075
Education				
Primary School and Below	ref.		ref.	
Junior High School	0.287 (0.178 0.463)	<0.001	0.551 (0.301 1.012)	0.055
High School/Technical School	0.276 (0.174 0.439)	<0.001	0.716 (0.376 1.361)	0.308
College/Bachelor's and Above	0.284 (0.170 0.474)	<0.001	1.798 (0.733 4.409)	0.200
Work and living conditions				
Shift work or night shift required	0.494 (0.258 0.946)	0.033	1.040 (0.442 2.447)	0.928
Daily working hours exceed 8	0.256 (0.15 0.432)	<0.001	0.572 (0.285 1.148)	0.116
Work or living environment with noise interference	0.326 (0.130 0.814)	0.016	0.450 (0.142 1.431)	0.176
None of the above	ref.		ref.	
Hypertension history, years				
≤5	ref.		ref.	
>5	2.653 (1.857 3.789)	<0.001	0.481 (0.121 1.916)	0.299
Family history of hypertension				
Yes	ref.		ref.	
No	0.548 (0.354 0.848)	0.007	1.012 (0.500 2.048)	0.974
Uncertain	0.864 (0.434 1.718)	0.676	1.982 (0.809 4.853)	0.134
Family history of hypertensive intracerebral hemorrhage				
Yes	ref.		ref.	
No	0.550 (0.365 0.830)	0.004	1.081 (0.590 1.981)	0.800
Uncertain	0.546 (0.358 0.832)	0.005	0.378 (0.218 0.656)	0.001
Had intracerebral hemorrhage in the past				
Yes	ref.			
No	0.756 (0.411 1.392)	0.370		
Diabetes				
Yes	ref.		ref.	
No	0.515 (0.335 0.793)	0.003	0.563 (0.308 1.026)	0.061
Coronary heart disease				
Yes	ref.		ref.	
No	0.279 (0.158 0.492)	<0.001	0.491 (0.233 1.032)	0.061
Smoking				
Yes	ref.		ref.	
No	12.856 (6.519 25.351)	<0.001	4.603 (1.954 10.845)	<0.001
Alcohol consumption				
Yes	ref.		ref.	
No	7.027 (4.174 11.552)	<0.001	3.522 (1.764 7.033)	<0.001
Duration of taking antihypertensive drugs, years				
<1	ref.		ref.	
1–3	0.418 (0.161 1.087)	0.074	0.604 (0.191 1.906)	0.390
3–5	1.611 (0.753 3.445)	0.219	2.344 (0.817 6.721)	0.113
>5	2.853 (1.901 4.281)	<0.001	3.275(0.804 13.348)	0.098

The multivariate analysis revealed a working or living environment with noise interference [OR = 13.191, 95%CI: (2.861–60.808); *P* = 0.001], no diabetes [OR = 1.929, 95%CI: (1.123–3.314); *P* = 0.017], no coronary heart disease [OR = 3.080, 95%CI: (1.573–6.028); *P* = 0.001], and taking antihypertensive drugs for 3–5 years [OR = 3.142, 95%CI: (1.133–8.708); *P* = 0.028] had independent associations with moderate and high compliance. Furthermore, higher education was associated with better compliance compared to graduating from primary school. Compliance was worse among those who were aware that they did not have a family history of hypertensive intracerebral hemorrhage [OR = 0.549, 95%CI: (0.320–0.942); *P* = 0.029], while compliance was better among those who were not aware of whether they had that or not [OR = 1.737, 95%CI: (1.004–3.005); *P* = 0.048] (Table 5).

**Table 5:** Analysis of factors affecting moderate/high compliance.

Variables	Univariate analysis		Multivariate analysis	
	OR (95%CI)	*P*	OR (95%CI)	*P*
Knowledge score	1.015 (0.960 1.073)	0.608		
Attitude score	1.043 (0.991 1.096)	0.106		
Practice score	0.988 (0.943 1.036)	0.623		
Gender				
Male	ref.			
Female	1.071 (0.743 1.546)	0.712		
Age	0.981 (0.970 0.992)	0.001	1.007 (0.986 1.029)	0.498
BMI				
Overweight	ref.		ref.	
Not overweight	1.098 (0.788 1.528)	0.582	0.916 (0.618 1.358)	0.664
Education				
Primary School and Below	ref.		ref.	
Junior High School	4.273 (2.647 6.897)	<0.001	4.188 (2.388 7.346)	<0.001
High School/Technical School	9.168 (5.575 15.075)	<0.001	6.846 (3.676 12.750)	<0.001
College/Bachelor's and Above	3.316 (1.996 5.111)	<0.001	4.181 (1.914 9.132)	<0.001
Work and living conditions				
Shift work or night shift required	0.754 (0.398 1.429)	0.386	0.897 (0.421 1.912)	0.779
Daily working hours exceed 8	1.011 (0.630 1.624)	0.963	1.053 (0.587 1.887)	0.863
Work or living environment with noise interference	7.913 (1.828 34.255)	0.006	13.191 (2.861 60.808)	0.001
None of the above	ref.		ref.	
Hypertension history, years				
≤ 5	ref.			
>5	1.068 (0.755 1.511)	0.710		
Family history of hypertension				
Yes	ref.			
No	0.667 (0.434 1.025)	0.065		
Uncertain	1.220 (0.600 2.483)	0.582		
Family history of hypertensive intracerebral hemorrhage				
Yes	ref.		ref.	
No	0.707 (0.472 1.058)	0.092	0.549 (0.320 0.942)	0.029
Uncertain	2.234 (1.445 3.455)	<0.001	1.737 (1.004 3.005)	0.048
Had intracerebral hemorrhage in the past				
Yes	ref.		ref.	
No	2.159 (1.166 3.996)	0.014	1.569 (0.732 3.360)	0.247
Diabetes				
Yes	ref.		ref.	
No	2.087 (1.372 3.176)	0.001	1.929 (1.123 3.314)	0.017
Coronary heart disease				
Yes	ref.		ref.	
No	3.982 (2.335 6.790)	<0.001	3.080 (1.573 6.028)	0.001
Smoking				
Yes	ref.			
No	1.043 (0.671 1.622)	0.851		
Alcohol consumption				
Yes	ref.			
No	1.068 (0.715 1.596)	0.748		
Duration of taking antihypertensive drugs, years				
<1	ref.			
1–3	0.661 (0.304 1.435)	0.295	0.655 (0.276 1.556)	0.338
3–5	3.276 (1.335 8.035)	0.010	3.142 (1.133 8.708)	0.028
>5	1.110 (0.750 1.642)	0.603	1.103(0.565 2.155)	0.774

**Figure 2:**
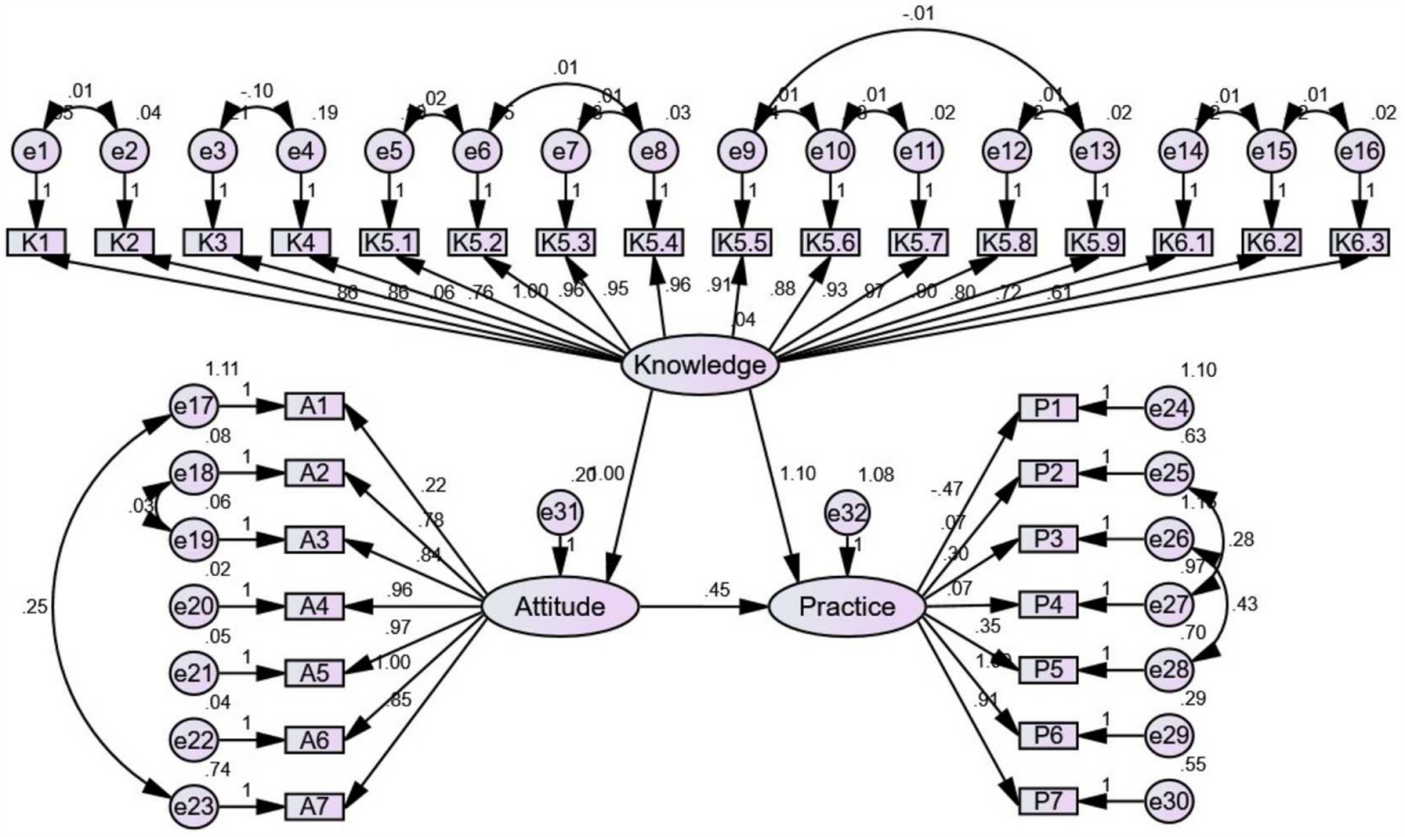
Structural equation model (SEM) for knowledge, attitudes, and practices (KAP).

SEM results revealed direct effects between knowledge and attitude (*β* = 0.999, *P* < 0.001), knowledge and practice (*β* = 1.103, *P* < 0.001), as well as attitude and practice (*β* = 0.452, *P* < 0.001) (Figure 2; Supplementary Table S4).

## Discussion

Hypertensive patients exhibited good knowledge, positive attitudes, but inactive preventive practices regarding intracerebral hemorrhage and poor medication adherence to hypertension. These findings emphasize the necessity of targeted interventions and educational programs to elevate awareness and encourage proactive health behaviors within this population.

Our investigation provided valuable insights into the KAP, and medication compliance among hypertensive patients, specifically in the context of preventing intracerebral hemorrhage. Gender differences were notable, with females displaying higher mean knowledge and practice scores compared to males, aligning with existing studies highlighting gender's influential role in health-related behaviors and outcomes (16, 17). Educational disparities were evident, with higher education levels correlating with greater knowledge and more favorable attitudes, consistent with literature emphasizing the positive link between education and health literacy (18). The impact of work and living conditions on KAP scores, particularly in shift work or night shifts, echoed prior research highlighting occupational factors’ influence on health behaviors (19). Family-centered findings revealed higher knowledge and practice scores among those with a family history of hypertensive intracerebral hemorrhage, consistent with studies emphasizing familial influences on health behaviors (20). SEM results indicating direct effects between knowledge, attitude, and practice aligned with the theoretical framework suggesting that enhanced knowledge positively influences attitudes and behaviors (21).

Additionally, our study unveiled unique associations, such as the independent association between working or living environments with noise interference and medication compliance. While some studies have examined the effects of environmental factors on hypertension, the specific correlation between noise interference and medication compliance in the context of hypertensive intracerebral hemorrhage patients warrants further investigation (22, 23). Understanding how noise interference affects medication adherence could inform targeted interventions to improve compliance in environments with heightened noise levels.

Furthermore, the contrasting findings related to awareness of a family history of hypertensive intracerebral hemorrhage and medication compliance raise intriguing questions. While individuals aware of not having a family history exhibited worse compliance, those uncertain about their family history showed better compliance. These results deviate from some studies suggesting that knowledge of a family history might enhance health-related behaviors (24). The nuanced relationship between awareness of family history and medication compliance merits in-depth exploration to elucidate the underlying factors contributing to these unexpected associations.

Our study offered a comprehensive overview of hypertensive patients’ knowledge dimensions regarding hypertensive intracerebral hemorrhage. Notably, high accuracy was observed across various knowledge dimensions, ranging from 68.83% to 96.67%. These results indicate a robust understanding among participants regarding the severe complications of hypertension, its diagnostic criteria, age of onset for hypertensive intracerebral hemorrhage, and appropriate responses to symptoms. However, the discrepancy in correct responses across different knowledge dimensions warrants attention, suggesting potential gaps that may need targeted interventions. This finding resonates with existing literature highlighting the need for continuous and specific education to address potential gaps in hypertensive patients’ understanding (25).

Responses to various attitude items among hypertensive patients revealed positive attitudes toward key aspects of hypertension control and prevention. Moreover, the acknowledgment of effective blood pressure control, lifestyle changes, and willingness to cooperate with doctors reflects a proactive stance toward hypertension management. However, a notable proportion expressed neutral or negative attitudes towards the importance of physical exercise, suggesting a potential area for intervention. This finding contrasts with extensive research highlighting the positive effect of regular physical activity on blood pressure control and overall cardiovascular health (26, 27). To address this, targeted improvement suggestions could involve implementing tailored education programs focusing on the benefits of physical exercise specifically for hypertensive patients. Incorporating personalized exercise plans into hypertension management strategies, considering patients’ preferences and physical capabilities, may enhance adherence to this crucial aspect of preventive care. These recommendations align with studies advocating for patient-centered approaches to promote positive health behaviors on hypertension (28, 29). Additionally, exploring and addressing potential barriers to adopting a positive attitude towards physical exercise, such as time constraints or perceived difficulty, may further enhance the effectiveness of interventions.

Responses in various practice dimensions among hypertensive patients provided crucial insights into their daily behaviors related to health management. A considerable proportion indicated suboptimal practices, particularly concerning blood pressure monitoring, dietary habits, and lifestyle choices. Notably, a significant number reported infrequent blood pressure monitoring, which is concerning given the importance of regular monitoring in hypertension management (30). Moreover, suboptimal dietary practices were evident, with a substantial percentage reporting infrequent consumption of fruits and vegetables rich in dietary fiber and high-frequency intake of dietary salt and sodium-containing condiments. These dietary patterns are recognized risk factors for hypertension and its complications (30). The high prevalence of frequent alcohol intake and smoking further compounds the risk, aligning with established associations between these behaviors and elevated blood pressure (31, 32). To address these challenges, targeted improvement suggestions could involve implementing comprehensive education programs emphasizing the importance of consistent blood pressure monitoring and providing practical tools or reminders to facilitate adherence. Dietary interventions could focus on culturally tailored strategies to increase the consumption of fruits, vegetables, and low-sodium alternatives, aligning with successful interventions documented in the literature (33, 34).

Despite the valuable insights gained, several limitations should be considered, including the cross-sectional design, use of self-reported data, and an uneven gender distribution among participants. The higher representation of female participants in this study reflects the epidemiological trend of hypertensive intracerebral hemorrhage being more prevalent in females, but it may still introduce potential selection bias. Additionally, the study did not account for variations in patients’ medication regimens, including the number and frequency of antihypertensive medications prescribed. These factors could influence medication adherence independently of KAP and may affect the interpretation of adherence-related findings. As the study was conducted in a hospital setting, there is a possibility of bias toward more favorable KAP outcomes, as hospital-attending patients are likely to have greater health awareness and motivation compared to the general population. Furthermore, the cross-sectional design limits the ability to infer causality and track changes over time. Future research could explore a longitudinal approach to better understand temporal relationships among KAP dimensions and medication adherence. Future research could address this limitation by conducting community-based studies to assess KAP among a broader and more diverse population. Further investigation could benefit from longitudinal studies and objective measures to enhance reliability. Addressing this gender imbalance through larger, multi-center studies could enhance the representativeness and generalizability of findings.

In conclusion, the research underscores the need for targeted interventions to enhance knowledge dissemination, foster positive attitudes, and promote active preventive practices among hypertensive patients. Strategies to improve medication adherence should be tailored based on identified factors, including education level and awareness of family history. These insights can inform more effective clinical practices and interventions to mitigate the risk of intracerebral hemorrhage among hypertensive individuals.

## Data Availability

The original contributions presented in the study are included in the article/Supplementary Material, further inquiries can be directed to the corresponding author.
