# Supplementary material for: Knowledge, attitudes, and practices regarding the prevention of intracerebral hemorrhage among hypertensive patients
Source: Front Cardiovasc Med. 2025 Jun 11;12:1361273. doi: 10.3389/fcvm.2025.1361273 (PMC12187756; doi:10.3389/fcvm.2025.1361273)
Supplement: Supplementary file 1 [file Table1.doc]

Supplementary Table 1: Responses to Various Knowledge Items

| **Items, n (%)** | **Correct** |
| --- | --- |
| **1. hypertensive intracerebral hemorrhage is a relatively severe complication of hypertension, primarily resulting from a sudden elevation in blood pressure leading to blood vessel rupture.** | 545(90.83) |
| **2. Hypertension is typically defined as a condition where a patient registers a systolic blood pressure of 140mmHg or higher, or a diastolic blood pressure of 90mmHg or higher, without the use of any antihypertensive medication.** | 551(91.83) |
| **3. The age of onset for hypertensive intracerebral hemorrhage is predominantly above 50 years.** | 177(29.50) |
| **4. hypertensive intracerebral hemorrhage often occurs during periods of rest and low physical activity.** | 413(68.83) |
| **5. Risk factors for hypertensive intracerebral hemorrhage include** |  |
| Hyperglycaemia | 496(82.67) |
| Hyperlipidemia | 541(90.17) |
| Obesity | 556(92.67) |
| High cholesterol | 552(92.00) |
| Smoking | 550(91.67) |
| Alcohol consumption | 561(93.50) |
| High-salt diet | 566(94.33) |
| Irregular use of antihypertensive medications | 559(93.17) |
| Prolonged periods of high stress and anxiety | 566(94.33) |
| **6. Hypertensive patients experiencing symptoms such as headache, nausea, and vomiting should take the following steps:** |  |
| Immediately cease physical activity and rest. | 570(95.00) |
| Check blood pressure, if it surpasses the normal range, adhere to the doctor's recommendations for taking antihypertensive medication. | 573(95.50) |
| If symptoms persist or worsen, seek prompt medical attention. | 580(96.67) |

Supplementary Table 2: Responses to Various Attitude Items

| **Item, n (%)** | **Strongly Agree** | **Agree** | **Neutral** | **Disagree** | **Strongly Disagree** |
| --- | --- | --- | --- | --- | --- |
| **1. Do you believe hypertensive patients should measure their blood pressure daily?** | 188(31.33) | 235(39.17) | 88(14.67) | 78(13.00) | 11(1.83) |
| **2. Do you believe hypertensive patients should regularly seek medical attention and adhere to prescribed medication schedules?** | 473(78.83) | 118(19.67) | 8(1.33) | 0 | 1(0.17) |
| **3. Do you think maintaining stable emotions and avoiding mental stress can be beneficial in preventing intracerebral hemorrhage?** | 478(79.67) | 111(18.50) | 9(1.50) | 1(0.17) | 1(0.17) |
| **4. Do you consider effective blood pressure control to be highly important in preventing hypertensive intracerebral hemorrhage?** | 474(79.00) | 117(19.50) | 7(1.17) | 0 | 2(0.33) |
| **5. Do you believe lifestyle changes such as limiting alcohol intake, quitting smoking, weight management, and regular exercise can help prevent intracerebral hemorrhage?** | 472(78.67) | 113(18.83) | 12(2.00) | 1(0.17) | 2(0.33) |
| **6. Are you willing to actively cooperate with your doctor's treatment plan in the process of controlling hypertension?** | 471(78.50) | 117(19.50) | 8(1.33) | 1(0.17) | 3(0.50) |
| **7. Do you acknowledge the importance of physical exercise for overall health and the prevention of hypertensive intracerebral hemorrhage?** | 401(66.83) | 106(17.67) | 48(8.00) | 39(6.50) | 6(1.00) |

Supplementary Table 3: Responses to Various Practice Items

| **Items, n (%)** | **Always** | **Often** | **Sometimes** | **Occasionally** | **Never** |
| --- | --- | --- | --- | --- | --- |
| **1. The frequency of your blood pressure monitoring.** | 88(14.67) | 129(21.50) | 76(12.67) | 295(49.17) | 12(2.00) |
| **2. The frequency of consuming fruits and vegetables rich in dietary fiber in your daily life.** | 204(34.00) | 318(53.00) | 47(7.83) | 27(4.50) | 4(0.67) |
| **3. The frequency of consuming dietary salt and sodium-containing condiments (such as soy sauce, sauces, oyster sauce, chicken essence, monosodium glutamate, etc.) in your daily diet.** | 71(11.83) | 220(36.67) | 77(12.83) | 217(36.17) | 15(2.50) |
| **4. The frequency of consuming milk in your daily life.** | 89(14.83) | 324(54.00) | 93(15.50) | 74(12.33) | 20(3.33) |
| **5. The frequency of consuming preserved foods (smoked meat, pickled vegetables, salted meat, salted duck eggs) in your daily life.** | 31(5.17) | 59(9.83) | 188(31.33) | 290(48.33) | 32(5.33) |
| **6. The frequency of alcohol intake in your daily life.** | 26(4.33) | 71(11.83) | 38(6.33) | 90(15.00) | 375(62.50) |
| **7. The frequency of smoking in your daily life.** | 30(5.00) | 65(10.83) | 22(3.67) | 26(4.33) | 457(76.17) |

Supplementary Table 4: SEM Results

|  |  |  | Estimate | P |
| --- | --- | --- | --- | --- |
| Attitude | <--- | Knowledge | 0.999 | <0.001 |
| Practice | <--- | Attitude | 0.452 | <0.001 |
| Practice | <--- | Knowledge | 1.103 | <0.001 |
